# Using propensity score matching analysis to compare between cardiometabolic risk factors and physical activity type in Korean adults: findings from a nationwide population-based survey

**DOI:** 10.1186/s12889-024-18567-x

**Published:** 2024-04-26

**Authors:** Min-Hyo Kim, Ji-Won Lee, John A. Linton, Yaeji Lee, Youhyun Song

**Affiliations:** 1 Department of Family Medicine, Severance Hospital, Yonsei University College of Medicine, 03722 Seoul, Republic of Korea; 2 Institute for Innovation in Digital Healthcare, Yonsei University, 03722 Seoul, Republic of Korea; 3 International Health Care Center, Severance Hospital, Yonsei University Health System, 03722 Seoul, Republic of Korea; 4 Department of Biostatistics and Computing, Yonsei University, 03722 Seoul, Republic of Korea; 5 Healthcare Research Team, Health Promotion Center, Gangnam Severance Hospital, Yonsei University College of Medicine, 06273 Seoul, Republic of Korea

**Keywords:** Aerobic exercise, Cardiovascular risk factors, Insulin resistance, Metabolic syndrome, Resistance exercise

## Abstract

**Purpose:**

We aimed to assess the effects of different exercise modalities on cardiometabolic risk factors within a comprehensive, representative sample of the Korean population.

**Methods:**

We categorized 13,971 adult participants into aerobic exercise (AE), resistance exercise (RE), combined aerobic and resistance exercise (TE), insufficient exercise, and inactive groups. Multivariable regressions were conducted to compare the incidence of chronic diseases across the groups before and after propensity score matching (PSM).

**Results:**

The TE and RE groups had significantly lower waist circumference (WC), mean blood pressure (BP), glucose and insulin-related indices, and white blood cell count (WBC) measures, with TE showing the most significant differences. The TE group had significantly lower triglyceride levels and higher high-density lipoprotein-cholesterol levels. Post-PSM, the TE group had the lowest risk for metabolic syndrome, hypertension, and diabetes, closely followed by the RE group when compared with the inactive group. In a subgroup analysis, RE consistently exhibited benefits including lower body mass index, WC, BP, total cholesterol, glucose and insulin-related indices, and WBC count when compared with AE. RE may be associated with reduced incidence of cardiometabolic diseases compared to AE alone.

**Conclusion:**

TE appears to be associated with significant reduction in cardiometabolic risk in Korean adults. RE possibly provides a more favorable cardiometabolic effect than AE.

## Introduction

In a recent global status report on chronic diseases, the World Health Organization (WHO) stated that cardiovascular disease (CVD), type 2 diabetes mellitus (DM), and obesity collectively account for approximately two-thirds of all global deaths [1]. Cardiometabolic syndrome refers to a group of metabolic abnormalities which are risk factors for cardiovascular disease. Cardiometabolic syndrome consists of abdominal obesity, high blood pressure, high blood sugar, high serum triglyceride (TG), and low serum high density lipoprotein (HDL) level [2]. To prevent these metabolic complications, regular physical activity (PA) is widely recognized as a vital component of a “healthy” lifestyle [3]. In contrast, sedentary behavior has been associated with adverse health outcomes including higher incidences of CVD, DM, and increased all-cause mortality [4].

PA can largely be divided into aerobic exercise (AE) and resistance exercise (RE). AE has been shown to contribute to improvements in blood lipids, blood pressure (BP), and cardiorespiratory fitness [5]. RE has positive effects on CVD risk factors, such as glucose metabolism, insulin sensitivity, and muscular strength and mass [5]. As a result, the WHO recommends both types of PA; and that adults aged 18–64 years should engage in moderate-intensity aerobic activity for at least 150 min per week, or vigorous-intensity exercise for at least 75 min per week, in addition to muscle-strengthening resistance exercises at least 2 days per week [4].

Despite numerous studies highlighting the benefits of generalized PA, there is limited evidence regarding the most effective type of exercise for preventing metabolic complications. To address this gap, we aimed to compare the cardiometabolic effects of AE, RE, and a combination of both (aerobic plus resistance, total exercise, i.e., TE) using a large-scale, representative sample of the general Korean population. We utilized various clinical indices including serum chemistry, WBC count, anthropometric measures, and glucose-metabolism related markers as well as cardiometabolic disease prevalence to identify these effects.

## Methods

### Study population

This study analyzed pooled data from the 2019–2021 8th Korean National Health and Nutrition Examination Survey (KNHANES). The KNHANES, conducted by the Korean Ministry of Health and Welfare, is a survey that focuses on non-institutionalized Korean residents within Korea. The survey employs a multistage, clustered probability design for sampling. The survey questions were developed by the Korea Institute for Health and Social Affairs and the Korea Centers for Disease Control (KCDC), with approval from the KCDC’s ethics committee. Comprehensive information concerning the KNHANES is available on their website: https://knhanes.kdca.go.kr/knhanes/eng/index.do.

**Fig. 1:**
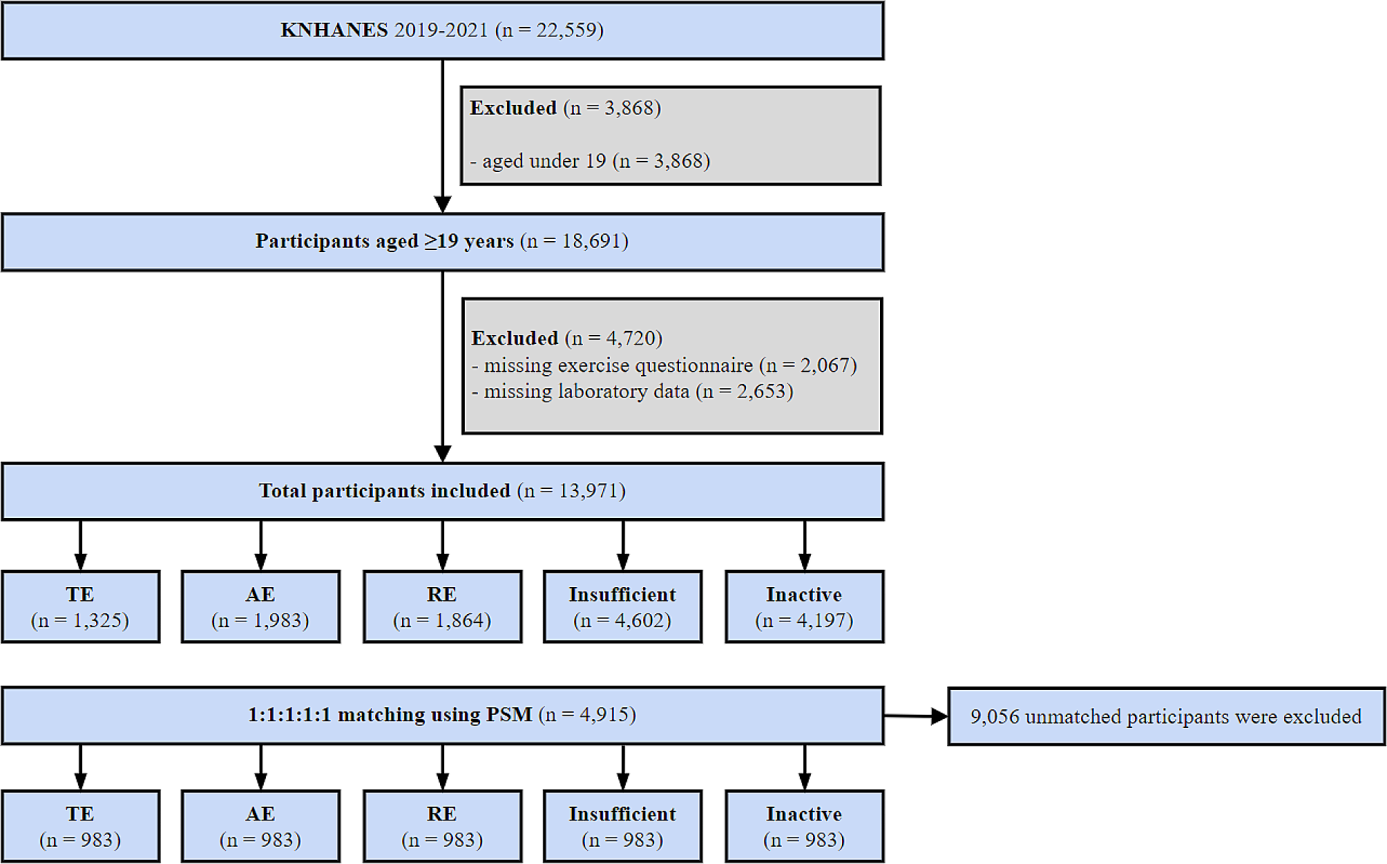
Flow chart of study population. KNHANES, 2019–2021 8th Korean National Health and Nutrition Examination Survey; insufficient exercise; inactive exercise; AE, aerobic exercise; RE, resistance exercise; TE, total exercise; PSM, Propensity score matching

The participants involved in this study were Korean adults aged ≥ 19 years. Initially, 18,691 adults participated in a health interview survey. We subsequently excluded individuals with missing data from the exercise questionnaire (*n* = 2,067) and those with missing laboratory data (*n* = 2,653), resulting in a final analysis cohort of 13,971 eligible participants (Fig. 1).

All eligible participants provided written informed consent to participate. This study adhered to the 1975 Declaration of Helsinki’s ethical guidelines and received approval from the Institutional Review Board (IRB) of Severance Hospital (IRB number 4-2023-0877).

### Anthropometric and laboratory measurements

Demographic, socioeconomic, and lifestyle data were collected using self-reported questionnaires.

Educational levels were categorized as follows: lower than elementary school, middle school, high school, and college or higher. Household income was determined using standardized criteria based on sex and 5-year age brackets, which were compared with standard income levels for Korean citizens. Monthly household income was calculated based on equalized income (total household income divided by the square root of the number of household members), and subsequently divided into quartiles. Smoking status was determined in accordance with respondents’ smoking history: those who had never smoked (or those who had smoked less than five packets of cigarettes but who did not smoke now), former smokers (those who had previously smoked more than five packets of cigarettes but were not currently smoking), and current smokers (those with a current smoking habit). A participant who consumed alcohol at least once a month was defined as being a current drinker.

Systolic BP (SBP) and diastolic BP (DBP) were determined through averaging the last two of three measurements with the patient seated. Mean arterial pressure was calculated using the DBP + 1/3(SBP − DBP) formula. Body weight and height were precisely measured to the nearest 0.1 kg and 0.001 m, respectively. Body mass index (BMI) was calculated as weight divided by the square of height (kg/m^2^). Obesity was categorized into normal, overweight, and obese groups, based on Asia Pacific region criteria [6]. Waist circumference (WC) was measured at the midpoint between the lowest rib and the iliac crest. Abdominal obesity was defined as a WC ≥ 90 cm in men and ≥ 85 cm in women, using Korean-specific criteria for abdominal obesity [7].

Blood samples were taken after participants had fasted for at least 8 h. Fasting glucose, total cholesterol, triglyceride (TG), high-density lipoprotein cholesterol (HDL-C), low-density lipoprotein cholesterol (LDL-C), aspartate aminotransferase (AST), and alanine aminotransferase (ALT) levels were measured using a Hitachi Automatic Analyzer 7600 (Hitachi). The white blood cell (WBC) count was measured using laser flow cytometry (XN9000 Sysmex, Japan). Fasting insulin was assessed using an electrochemiluminescence immunoassay (ECLIA; Roche, Germany) with modular E801 (Roche, Germany). Insulin resistance was evaluated using homeostasis model assessment-estimated insulin resistance (HOMA-IR) and triglyceride glucose (TyG) indexes, with the following Equations (1 and 2):

1 $$\eqalign{ HOMA - IR, & fasting{\mkern 1mu} insulin(\mu U/mL) \times \cr & fasting{\mkern 1mu} glucose\left( {mg/dL} \right)/405 \cr} $$

2 $$\eqalign{& TyG{\mkern 1mu} index,Ln \cr & \left[ {triglyceride\left( {mg/dl} \right) \times fasting{\mkern 1mu} glucose\left( {mg/dL} \right)/2} \right] \cr} $$

Metabolic syndrome (MetS) was defined as having at least three of the following criteria: (i) abdominal obesity; (ii) serum TGs, ≥ 150 mg/dL or taking lipid-lowering medication; (iii) low serum HDL-cholesterol (< 40 mg/dL for men; <50 mg/dL for women); (iv) SBP, ≥ 130 mmHg; DBP, ≥ 85 mmHg; or taking anti-hypertensive medication, and; (v) fasting plasma glucose, ≥ 100 mg/dL; taking anti-diabetic medication; or undergoing insulin therapy.

Hypertension (HTN) was defined as: (i) SBP, ≥ 140 mmHg; (ii) DBP, ≥ 90 mmHg; or (iii) taking anti-hypertensive medication. DM was diagnosed if one of the following were met: (i) fasting glucose level, ≥ 126 mg/dL; (ii) plasma glucose, ≥ 200 mg/dL following a 75 g oral glucose tolerance test; (iii) HbA1c, ≥ 6.5%; (iv) taking anti-DM medication; or (v) undergoing insulin therapy. Dyslipidemia was diagnosed if one of the following criteria were met: (i) total cholesterol, ≥ 240 mg/dL; (ii) HDL cholesterol, ≤ 40 mg/dL; (iii) LDL-cholesterol, ≥ 160 mg/dL; or (iv) TGs, ≥ 200 mg/dL. The detailed data resource profile and the methods and devices used in measurements are described elsewhere [8].

### Classification of the PA group

PA levels were assessed using the Global Physical Activity Questionnaire, which has been translated into Korean and validated for reliability [9]. The survey comprises 16 questions across work, transport, leisure-time PA, and sedentary behavior domains.

Each domain (work and leisure, 6 questions; transport, 3 questions; sedentary, 1 question) contributes to the total PA calculation. Descriptive analysis presents domain-specific PA in terms of min/week. In the primary study, we employed metabolic equivalents task (METs)*min/week (8 METs for moderate PA, 16 METs for vigorous PA) to assess PA intensity. Additionally, RE participation was assessed according to weekly frequency.

We divided PA into five groups: TE, AE, RE, Insufficient, and Inactive. To accord with WHO recommendations [1], adults were classified into the AE group if they attained ≥ 1200 METs*min/week [10]. Individuals engaging in moderate- or higher-intensity muscle-strengthening activities for at least 2 days a week were categorized into the RE group. The TE group comprised participants who sufficiently engaged in both aerobic exercise and strength training according to the aforementioned criteria. The Insufficient group comprised participants who engaged in aerobic exercise and/or resistance exercise only to an insufficient level (aerobic exercise less than 1200 METs*min/week and/or resistance exercise less than twice a week). Finally, the Inactive group included participants who did not engage in any measurable PA by the modalities mentioned.

### Statistical analysis

Pre-matching data are presented as means ± standard error (SE) or as prevalence (%). Sampling weights were used to account for complex sampling. The characteristics of the exercise groups were compared using analysis of variance or a Student’s *t*-test for continuous data and a Pearson’s chi-square test.

We conducted propensity score matching (PSM), employing a 1:1:1:1:1 matching ratio to address potential confounding effects of age and sex using an exact-matching algorithm. Propensity score matching which is a method that equalizes experimental and control groups on an inclusive set of estimated variables in observational studies, is a single mathematical value of probability which abstracts the selected variables, using logistic regression analysis [11]. The propensity score matching is considered as useful tool when analyzing cross-sectional data and selected in numerous recent articles [12, 13]. The propensity score signifies the likelihood of being subjected to a specific treatment based on the observed covariates. Standardized mean differences (SMDs) were calculated to evaluate the balance of covariates before and after PSM implementation. An SMD value < 0.1 denoted a balanced distribution of data.

Multivariate regressions were performed to compare the cardiometabolic risk factors (anthropometric findings, lab results, glucose-metabolism related indices, and disease prevalence) among the PA groups after adjusting for alcohol consumption, smoking history, educational status, and household income status. The results are presented as coefficients and 95% confidence intervals (CIs). Dependent variables such as MetS, HTN, DM, and dyslipidemia were modeled through multiple logistic regression, and the results were reported as odds ratios (ORs) and 95% CIs. Furthermore, a subgroup analysis was performed to directly compare the AE group with the RE group.

*P*-values < 0.05 were considered statistically significant. All statistical analyses were conducted using R version 4.3.0 (R Foundation for Statistical Computing, Vienna, Austria, http://www. R-project. org/) software.

## Results

Table 1 shows the general characteristics of the study population. Of 13,971 participants in total, 49.2% were men (average age, 48.2 years). When divided into five groups according to their PA status prior to matching, most participants were in the insufficient group (*n* = 4,602, 32.94%), followed by the inactive (*n* = 4,197, 30.04%), AE (*n* = 1,983, 14.19%), RE (*n* = 1,864, 13.34%), and TE (*n* = 1,325, 9.48%) groups. All socio-economic and lifestyle variables differed significantly between the five groups, which were later adjusted for the study analysis.

**Table 1:** Clinical characteristics according to exercise type pre- and post-PSM

Variable	Pre-PSM							Post-PSM							
	Overall	TE group	AE group	RE group	Insufficient	Inactive	p-value	Overall	TE group	AE group	RE group	Insufficient		Inactive	*p*-value
Unweighted N	13,971	1325	1983	1864	4602	4197		4915	983	983	983		983	983	
Age (years)	48.20 ± 16.43	41.70 ± 16.10	45.36 ± 15.22	49.11 ± 16.72	47.96 ± 16.26	52.02 ± 16.15	< 0.001**	49.23 ± 15.79	49.23 ± 15.80	49.23 ± 15.80	49.23 ± 15.80		49.23 ± 15.80	49.23 ± 15.80	> 0.999
Sex (%)							< 0.001**								> 0.999
Male	49.2	68.1	49.7	63.8	40.2	45.3		56.8	56.8	56.8	56.8		56.8	56.8	
Female	50.8	31.9	50.3	36.2	59.8	54.7		43.2	43.2	43.2	43.2		43.2	43.2	
Educational status (%)							< 0.001**								< 0.001**
Elementary school	12	3.7	7	8.8	12.2	19.3		9.8	6.2	8.5	9.1		10.8	14.5	
Middle school	7.8	4.8	6.3	8	7.8	9.8		8.9	7	8.4	8.2		10	11	
High school	36.9	39.6	37.6	37.3	37.1	34.9		36.5	37	36.3	36.1		36.8	36.4	
College or University	43.3	51.9	49.1	46	42.9	36		44.7	49.7	46.7	46.6		42.4	38	
Alcohol consumption (yes, %)	55.4	66	60.1	60.2	52.7	49.6	< 0.001**	59.6	63	60.8	59.7		57.2	57.1	0.036*
Smoking history (%)							< 0.001**								< 0.001**
No	57.8	49.6	57.1	50.1	63.3	58.8		51.8	53.2	52	52.2		51.8	49.7	
Current	18.2	19.6	19.3	17	16.5	19.9		19.3	15.1	20.2	16.3		20.5	24.5	
Former	23.9	30.8	23.7	33	20.3	21.4		28.9	31.7	27.8	31.5		27.7	25.7	
Household income status (%)							< 0.001**								< 0.001**
Low	14.1	7.5	10.8	13.2	14.4	18.6		13	10.1	12.8	11.9		15.3	15.2	
Mid-low	23.1	20	21.9	21.7	24.7	23.7		22.8	19.9	22.6	22.6		23.2	25.9	
Mid-high	29.3	30.4	30.1	27.8	29.3	29.2		29.9	29.6	29.7	29.6		31.2	29.5	
High	33.4	42.1	37.2	37.2	31.6	28.4		34.2	40.4	34.9	35.9		30.3	29.4	
Body mass index (%)							< 0.001**								< 0.001**
Underweight (< 18.5)	4	2.4	3.6	3	5.2	4		3.5	1.7	3.5	3.9		3.6	5	
Normal (≥ 18.5 and < 25)	58.8	61.4	55.4	61.8	59	57.9		59.7	63.2	56.2	64.3		58.3	56.6	
Obese (≥ 25)	37.2	36.3	41.1	35.2	35.8	38.1		36.8	35.1	40.4	31.8		38.1	38.5	
WC (cm)	84.19 ± 10.86	83.46 ± 10.21	84.37 ± 10.96	84.01 ± 10.32	83.55 ± 11.14	85.23 ± 10.88	< 0.001**	84.45 ± 10.63	83.34 ± 9.61	85.05 ± 10.68	83.16 ± 10.49		85.31 ± 11.06	85.39 ± 11.03	< 0.001**
Mean BP (mmHg)	89.79 ± 10.77	88.94 ± 10.22	89.73 ± 10.88	89.34 ± 10.01	89.65 ± 11.09	90.54 ± 10.85	< 0.001**	90.27 ± 10.53	89.53 ± 10.24	90.59 ± 10.78	89.14 ± 10.05		90.88 ± 11.01	91.20 ± 10.41	< 0.001**
Total cholesterol (mg/dL)	193.05 ± 38.66	193.61 ± 37.27	195.59 ± 38.59	191.02 ± 37.26	193.44 ± 38.26	192.00 ± 40.23	0.001**	193.24 ± 38.65	194.02 ± 38.90	194.88 ± 39.49	193.09 ± 37.11		191.48 ± 38.68	192.71 ± 38.99	0.354
TG (mg/dL)	131.10 ± 106.97	119.52 ± 117.23	131.58 ± 123.47	127.73 ± 107.46	129.89 ± 94.78	138.34 ± 106.24	< 0.001**	131.29 ± 104.66	117.74 ± 92.66	131.62 ± 104.59	131.42 ± 129.07		133.86 ± 87.64	141.82 ± 103.18	< 0.001**
HDL-cholesterol (mg/dL)	52.25 ± 12.83	54.02 ± 13.18	53.22 ± 13.21	51.95 ± 12.86	52.49 ± 12.50	50.92 ± 12.72	< 0.001**	52.49 ± 13.07	54.60 ± 13.84	52.94 ± 13.30	52.70 ± 13.03		51.15 ± 12.09	51.04 ± 12.73	< 0.001**
LDL-cholesterol (mg/dL)	116.14 ± 34.55	117.17 ± 32.91	117.64 ± 34.00	114.98 ± 33.27	116.45 ± 34.90	115.15 ± 35.59	0.027*	116.03 ± 34.61	116.80 ± 34.52	116.96 ± 35.11	115.92 ± 32.88		115.08 ± 35.56	115.39 ± 34.94	0.684
Glucose (mg/dL)	100.79 ± 22.35	97.47 ± 19.15	99.80 ± 21.52	99.52 ± 18.90	100.92 ± 23.02	103.06 ± 24.33	< 0.001**	101.52 ± 22.28	100.36 ± 21.75	101.55 ± 22.59	100.17 ± 20.33		103.03 ± 23.76	102.47 ± 22.72	0.014*
Insulin (IU/L)	9.07 ± 6.97	7.99 ± 5.64	9.06 ± 6.42	8.57 ± 6.40	9.23 ± 6.82	9.55 ± 8.00	< 0.001**	8.64 ± 6.41	7.65 ± 5.35	8.71 ± 6.18	8.28 ± 5.72		9.36 ± 7.91	9.20 ± 6.44	< 0.001**
HOMR-IR	2.34 ± 2.20	1.98 ± 1.84	2.30 ± 1.91	2.18 ± 1.89	2.38 ± 2.19	2.53 ± 2.56	< 0.001**	2.25 ± 2.17	1.96 ± 1.93	2.23 ± 1.80	2.13 ± 1.96		2.49 ± 2.96	2.40 ± 1.95	< 0.001**
HbA1c (%)	5.77 ± 0.82	5.61 ± 0.68	5.72 ± 0.79	5.71 ± 0.69	5.78 ± 0.87	5.87 ± 0.87	< 0.001**	5.78 ± 0.81	5.72 ± 0.77	5.80 ± 0.79	5.73 ± 0.73		5.83 ± 0.89	5.82 ± 0.86	0.007**
TyG Index	8.59 ± 0.66	8.44 ± 0.67	8.57 ± 0.66	8.55 ± 0.65	8.59 ± 0.64	8.66 ± 0.66	< 0.001**	8.60 ± 0.66	8.49 ± 0.65	8.61 ± 0.64	8.56 ± 0.68		8.66 ± 0.63	8.69 ± 0.67	< 0.001**
AST (IU/L)	24.82 ± 14.36	25.99 ± 16.63	24.82 ± 13.60	25.88 ± 20.94	24.19 ± 12.35	24.58 ± 11.83	< 0.001**	25.26 ± 16.22	25.93 ± 16.72	24.85 ± 11.82	25.56 ± 20.90		25.41 ± 17.66	24.54 ± 12.12	0.324
ALT (IU/L)	24.53 ± 20.39	25.69 ± 21.48	24.83 ± 19.38	24.88 ± 21.74	23.85 ± 19.78	24.57 ± 20.48	0.026*	24.71 ± 20.53	24.53 ± 19.86	23.96 ± 16.76	24.01 ± 21.16		25.49 ± 20.60	25.56 ± 23.67	0.231
WBC (× 10^3^/µL)	6.17 ± 1.68	6.08 ± 1.67	6.17 ± 1.64	6.03 ± 1.60	6.17 ± 1.69	6.26 ± 1.70	< 0.001**	6.13 ± 1.68	5.89 ± 1.61	6.18 ± 1.64	5.90 ± 1.56		6.28 ± 1.74	6.41 ± 1.77	< 0.001**
MetS (yes, %)	27.8	18.6	26.1	24.1	28.3	33.3	< 0.001**	27.9	22.2	28.9	23.1		31.8	33.6	< 0.001**
HTN (yes, %)	54.4	49.5	51.8	54.6	52.5	59.7	< 0.001**	57	53.3	59.6	53.1		59.5	59.7	< 0.001**
DM (yes, %)	53.4	40.7	50.9	52.2	52.9	60.8	< 0.001**	55.5	52	56.9	53.3		57.4	58	0.022*
Dyslipidemia (yes, %)	34.9	27.5	33.5	32.9	35	39.4	< 0.001**	35.4	33.4	38.1	32.3		36	37.2	0.031*

*Note* All continuous data are presented as means ± standard deviation (SD); All categorical data are presented as percentage (%); †Analysis of variance (ANOVA) or Pearson’s chi-square test were used to compare the exercise groups; * *p* < 0.05; ** *p* < 0.01

*Abbreviation* AE, aerobic exercise; ALT, alanine aminotransferase; AST, aspartate aminotransferase; BMI, body mass index; BP, blood pressure; CE, complex exercise; DM, diabetes mellitus; HDL, high density lipoprotein; HOMA-IR, homeostasis model assessment-estimated insulin resistance; HTN, hypertension; LDL, low-density lipoprotein; MetS, metabolic syndrome; PSM, propensity score matching; RE, resistance exercise; TG, triglyceride; TyG index, triglyceride and glucose index; TE, total exercise; WBC, white blood cell; WC, waist circumference

After matching, each group consisted of 983 participants (males, 56.8%; average age, 49.2 years). WC and the prevalence of obesity according to BMI were both lowest in the RE group followed by TE group. Mean BP and the prevalence of HTN was also lowest in RE group, closely followed by TE group. Total cholesterol and LDL-C levels did not differ significantly between the five groups; however, TG was significantly lower, and HDL-C was significantly higher in the TE group compared with the other PA groups. The Inactive group showed the highest TG and lowest HDL-C levels among the five groups. Fasting glucose, HbA1c, serum insulin, HOMA-IR and TyG index uniformly showed that the TE group significantly had the lowest mean values compared with other groups, closely followed by RE, then AE group. The WBC count also followed this trend. Accordingly, the prevalence of DM and MetS were lowest in the TE group, followed by RE, then AE groups.

Table 2 shows the regression coefficients between the five PA groups after PSM. All variables with the exception of total cholesterol and LDL-C showed the most decreased or desirable values in TE group, closely followed by RE group, then AE group. WC, mean BP, WBC count, and some glucose-related indices (fasting glucose, HOMA-IR, and HbA1C) were found to have insignificant differences between TE and RE groups. Total cholesterol and LDL-C showed no significant differences between the five groups; however, HDL-C levels were higher in all exercise groups compared with the insufficient and inactive groups and highest in the TE group. TG levels were significantly decreased (–15.77 mg/dL &–-23.06 mg/dL) in the TE group compared to the insufficient and inactive groups; while AE and RE showed decreased TG level compared with the inactive groups only.

**Table 2:** Comparison of clinical characteristics between the AE, RE, non-AE and RE, and TE groups post-PSM

Variable	TE group		AE only group		RE only group		Insufficient		Inactive		*p*-value
	Coefficient (95% CI)	*p*-value	Coefficient (95% CI)	*p*-value	Coefficient (95% CI)	*p*-value	Coefficient (95% CI)	*p*-value	Coefficient (95% CI)	*p*-value	
BMI (kg/m^2^)	Ref	Ref	0.262 (-0.054, 0.579)	0.105	-0.350 (-0.666, -0.033)	0.03*	0.215 (-0.103, 0.533)	0.185	0.096 (-0.224, 0.416)	0.558	0.001**
	-0.262 (-0.579, 0.054)	0.105	Ref	Ref	-0.612 (-0.929, -0.296)	< 0.001**	-0.047 (-0.364, 0.269)	0.77	-0.167 (-0.484, 0.151)	0.304	
	0.350 (0.033, 0.666)	0.03*	0.612 (0.296, 0.929)	< 0.001**	Ref	Ref	0.565 (0.248, 0.882)	< 0.001**	0.445 (0.127, 0.764)	0.006**	
	-0.215 (-0.533, 0.103)	0.185	0.047 (-0.269, 0.364)	0.77	-0.565 (-0.882, -0.248)	< 0.001**	Ref	Ref	-0.119 (-0.436, 0.197)	0.46	
	-0.096 (-0.416, 0.224)	0.558	0.167 (-0.151, 0.484)	0.304	-0.445 (-0.764, -0.127)	0.006**	0.119 (-0.197, 0.436)	0.46	Ref	Ref	
WC (cm)	Ref	Ref	1.660 (0.828, 2.492)	< 0.001**	-0.244 (-1.075, 0.587)	0.565	1.869 (1.034, 2.704)	< 0.001**	1.844 (1.004, 2.685)	< 0.001**	< 0.001**
	-1.660 (-2.492, -0.828)	< 0.001**	Ref	Ref	-1.904 (-2.735, -1.074)	< 0.001**	0.209 (-0.622, 1.040)	0.622	0.184 (-0.650, 1.019)	0.665	
	0.244 (-0.587, 1.075)	0.565	1.904 (1.074, 2.735)	< 0.001**	Ref	Ref	2.113 (1.282, 2.945)	< 0.001**	2.089 (1.253, 2.924)	< 0.001**	
	-1.869 (-2.704, -1.034)	< 0.001**	-0.209 (-1.040, 0.622)	0.622	-2.113 (-2.945, -1.282)	< 0.001**	Ref	Ref	-0.025 (-0.856, 0.807)	0.954	
	-1.844 (-2.685, -1.004)	< 0.001**	-0.184 (-1.019, 0.650)	0.665	-2.089 (-2.924, -1.253)	< 0.001**	0.025 (-0.807, 0.856)	0.954	Ref	Ref	
Mean BP (mmHg)	Ref	Ref	1.085 (0.197, 1.973)	0.017*	-0.359 (-1.246, 0.528)	0.427	1.374 (0.483, 2.265)	0.003**	1.676 (0.779, 2.573)	< 0.001**	< 0.001**
	-1.085 (-1.973, -0.197)	0.017*	Ref	Ref	-1.445 (-2.331, -0.558)	0.001**	0.289 (-0.598, 1.176)	0.523	0.591 (-0.299, 1.481)	0.193	
	0.359 (-0.528, 1.246)	0.427	1.445 (0.558, 2.331)	0.001**	Ref	Ref	1.733 (0.846, 2.621)	< 0.001**	2.036 (1.144, 2.927)	< 0.001**	
	-1.374 (-2.265, -0.483)	0.003**	-0.289 (-1.176, 0.598)	0.523	-1.733 (-2.621, -0.846)	< 0.001**	Ref	Ref	0.302 (-0.585, 1.190)	0.504	
	-1.676 (-2.573, -0.779)	< 0.001**	-0.591 (-1.481, 0.299)	0.193	-2.036 (-2.927, -1.144)	< 0.001**	-0.302 (-1.190, 0.585)	0.504	Ref	Ref	
Total cholesterol (mg/dL)	Ref	Ref	1.256 (-2.137, 4.650)	0.468	-0.438 (-3.828, 2.952)	0.8	-1.620 (-5.026, 1.786)	0.351	0.096 (-3.332, 3.524)	0.956	0.578
	-1.256 (-4.650, 2.137)	0.468	Ref	Ref	-1.694 (-5.082, 1.694)	0.327	-2.876 (-6.266, 0.514)	0.096	-1.160 (-4.563, 2.242)	0.504	
	0.438 (-2.952, 3.828)	0.8	1.694 (-1.694, 5.082)	0.327	Ref	Ref	-1.182 (-4.574, 2.211)	0.495	0.534 (-2.873, 3.941)	0.759	
	1.620 (-1.786, 5.026)	0.351	2.876 (-0.514, 6.266)	0.096	1.182 (-2.211, 4.574)	0.495	Ref	Ref	1.716 (-1.676, 5.108)	0.321	
	-0.096 (-3.524, 3.332)	0.956	1.160 (-2.242, 4.563)	0.504	-0.534 (-3.941, 2.873)	0.759	-1.716 (-5.108, 1.676)	0.321	Ref	Ref	
TG (mg/dL)	Ref	Ref	13.388 (4.367, 22.409)	0.004**	13.707 (4.693, 22.720)	0.003**	15.770 (6.715, 24.825)	< 0.001**	23.061 (13.947, 32.176)	< 0.001**	< 0.001**
	-13.388 (-22.409, -4.367)	0.004**	Ref	Ref	0.319 (-8.689, 9.326)	0.945	2.382 (-6.631, 11.395)	0.604	9.674 (0.628, 18.719)	0.036*	
	-13.707 (-22.720, -4.693)	0.003**	-0.319 (-9.326, 8.689)	0.945	Ref	Ref	2.063 (-6.956, 11.082)	0.654	9.355 (0.297, 18.412)	0.043*	
	-15.770 (-24.825, -6.715)	< 0.001**	-2.382 (-11.395, 6.631)	0.604	-2.063 (-11.082, 6.956)	0.654	Ref	Ref	7.292 (-1.727, 16.310)	0.113	
	-23.061 (-32.176, -13.947)	< 0.001**	-9.674 (-18.719, -0.628)	0.036*	-9.355 (-18.412, -0.297)	0.043*	-7.292 (-16.310, 1.727)	0.113	Ref	Ref	
HDL-cholesterol (mg/dL)	Ref	Ref	-1.453 (-2.511, -0.395)	0.007**	-1.675 (-2.732, -0.618)	0.002**	-2.997 (-4.059, -1.935)	< 0.001**	-2.990 (-4.059, -1.921)	< 0.001**	< 0.001**
	1.453 (0.395, 2.511)	0.007**	Ref	Ref	-0.222 (-1.278, 0.835)	0.681	-1.544 (-2.601, -0.487)	0.004**	-1.537 (-2.598, -0.476)	0.005**	
	1.675 (0.618, 2.732)	0.002**	0.222 (-0.835, 1.278)	0.681	Ref	Ref	-1.322 (-2.380, -0.265)	0.014*	-1.315 (-2.378, -0.253)	0.015*	
	2.997 (1.935, 4.059)	< 0.001**	1.544 (0.487, 2.601)	0.004**	1.322 (0.265, 2.380)	0.014*	Ref	Ref	0.007 (-1.051, 1.065)	0.989	
	2.990 (1.921, 4.059)	< 0.001**	1.537 (0.476, 2.598)	0.005**	1.315 (0.253, 2.378)	0.015*	-0.007 (-1.065, 1.051)	0.989	Ref	Ref	
LDL-cholesterol (mg/dL)	Ref	Ref	0.403 (-2.640, 3.447)	0.795	-0.636 (-3.677, 2.405)	0.682	-1.250 (-4.305, 1.805)	0.423	-0.482 (-3.558, 2.593)	0.758	0.858
	-0.403 (-3.447, 2.640)	0.795	Ref	Ref	-1.040 (-4.079, 1.999)	0.502	-1.653 (-4.694, 1.388)	0.287	-0.886 (-3.938, 2.166)	0.569	
	0.636 (-2.405, 3.677)	0.682	1.040 (-1.999, 4.079)	0.502	Ref	Ref	-0.614 (-3.657, 2.429)	0.693	0.154 (-2.902, 3.210)	0.921	
	1.250 (-1.805, 4.305)	0.423	1.653 (-1.388, 4.694)	0.287	0.614 (-2.429, 3.657)	0.693	Ref	Ref	0.768 (-2.275, 3.810)	0.621	
	0.482 (-2.593, 3.558)	0.758	0.886 (-2.166, 3.938)	0.569	-0.154 (-3.210, 2.902)	0.921	-0.768 (-3.810, 2.275)	0.621	Ref	Ref	
Glucose (mg/dL)	Ref	Ref	1.020 (-0.859, 2.898)	0.287	-0.249 (-2.126, 1.628)	0.795	2.464 (0.578, 4.350)	0.01*	1.762 (-0.136, 3.660)	0.069	0.023*
	-1.020 (-2.898, 0.859)	0.287	Ref	Ref	-1.269 (-3.145, 0.607)	0.185	1.444 (-0.432, 3.321)	0.131	0.743 (-1.141, 2.626)	0.44	
	0.249 (-1.628, 2.126)	0.795	1.269 (-0.607, 3.145)	0.185	Ref	Ref	2.713 (0.835, 4.591)	0.005**	2.011 (0.125, 3.898)	0.037*	
	-2.464 (-4.350, -0.578)	0.01*	-1.444 (-3.321, 0.432)	0.131	-2.713 (-4.591, -0.835)	0.005**	Ref	Ref	-0.702 (-2.580, 1.176)	0.464	
	-1.762 (-3.660, 0.136)	0.069	-0.743 (-2.626, 1.141)	0.44	-2.011 (-3.898, -0.125)	0.037*	0.702 (-1.176, 2.580)	0.464	Ref	Ref	
Insulin (IU/L)	Ref	Ref	1.035 (0.474, 1.596)	< 0.001**	0.570 (0.010, 1.130)	0.046*	1.622 (1.059, 2.184)	< 0.001**	1.438 (0.872, 2.005)	< 0.001**	< 0.001**
	-1.035 (-1.596, -0.474)	< 0.001**	Ref	Ref	-0.465 (-1.025, 0.095)	0.103	0.586 (0.026, 1.147)	0.04*	0.403 (-0.159, 0.965)	0.16	
	-0.570 (-1.130, -0.010)	0.046*	0.465 (-0.095, 1.025)	0.103	Ref	Ref	1.052 (0.491, 1.612)	< 0.001**	0.868 (0.305, 1.431)	0.003**	
	-1.622 (-2.184, -1.059)	< 0.001**	-0.586 (-1.147, -0.026)	0.04*	-1.052 (-1.612, -0.491)	< 0.001**	Ref	Ref	-0.183 (-0.744, 0.377)	0.521	
	-1.438 (-2.005, -0.872)	< 0.001**	-0.403 (-0.965, 0.159)	0.16	-0.868 (-1.431, -0.305)	0.003**	0.183 (-0.377, 0.744)	0.521	Ref	Ref	
HOMA-IR	Ref	Ref	0.260 (0.069, 0.450)	0.008**	0.156 (-0.035, 0.346)	0.109	0.499 (0.307, 0.690)	< 0.001**	0.402 (0.209, 0.594)	< 0.001**	< 0.001**
	-0.260 (-0.450, -0.069)	0.008**	Ref	Ref	-0.104 (-0.294, 0.086)	0.284	0.239 (0.048, 0.429)	0.014*	0.142 (-0.049, 0.333)	0.146	
	-0.156 (-0.346, 0.035)	0.109	0.104 (-0.086, 0.294)	0.284	Ref	Ref	0.343 (0.152, 0.534)	< 0.001**	0.246 (0.055, 0.437)	0.012*	
	-0.499 (-0.690, -0.307)	< 0.001**	-0.239 (-0.429, -0.048)	0.014*	-0.343 (-0.534, -0.152)	< 0.001**	Ref	Ref	-0.097 (-0.288, 0.094)	0.318	
	-0.402 (-0.594, -0.209)	< 0.001**	-0.142 (-0.333, 0.049)	0.146	-0.246 (-0.437, -0.055)	0.012*	0.097 (-0.094, 0.288)	0.318	Ref	Ref	
HbA1c (%)	Ref	Ref	0.061 (-0.007, 0.128)	0.077	-0.003 (-0.071, 0.064)	0.926	0.080 (0.012, 0.148)	0.021*	0.063 (-0.006, 0.131)	0.072	0.041*
	-0.061 (-0.128, 0.007)	0.077	Ref	Ref	-0.064 (-0.132, 0.003)	0.062	0.019 (-0.049, 0.086)	0.583	0.002 (-0.066, 0.069)	0.962	
	0.003 (-0.064, 0.071)	0.926	0.064 (-0.003, 0.132)	0.062	Ref	Ref	0.083 (0.015, 0.150)	0.016*	0.066 (-0.002, 0.134)	0.057	
	-0.080 (-0.148, -0.012)	0.021*	-0.019 (-0.086, 0.049)	0.583	-0.083 (-0.150, -0.015)	0.016*	Ref	Ref	-0.017 (-0.085, 0.050)	0.616	
	-0.063 (-0.131, 0.006)	0.072	-0.002 (-0.069, 0.066)	0.962	-0.066 (-0.134, 0.002)	0.057	0.017 (-0.050, 0.085)	0.616	Ref	Ref	
TyG index	Ref	Ref	0.117 (0.062, 0.171)	< 0.001**	0.068 (0.013, 0.122)	0.015*	0.170 (0.116, 0.225)	< 0.001**	0.192 (0.137, 0.247)	< 0.001**	< 0.001**
	-0.117 (-0.171, -0.062)	< 0.001**	Ref	Ref	-0.049 (-0.103, 0.005)	0.077	0.054 (-0.001, 0.108)	0.052	0.075 (0.020, 0.130)	0.007**	
	-0.068 (-0.122, -0.013)	0.015*	0.049 (-0.005, 0.103)	0.077	Ref	Ref	0.103 (0.048, 0.157)	< 0.001**	0.124 (0.069, 0.179)	< 0.001**	
	-0.170 (-0.225, -0.116)	< 0.001**	-0.054 (-0.108, 0.001)	0.052	-0.103 (-0.157, -0.048)	< 0.001**	Ref	Ref	0.021 (-0.033, 0.076)	0.444	
	-0.192 (-0.247, -0.137)	< 0.001**	-0.075 (-0.130, -0.020)	0.007**	-0.124 (-0.179, -0.069)	< 0.001**	-0.021 (-0.076, 0.033)	0.444	Ref	Ref	
AST (IU/L)	Ref	Ref	-1.129 (-2.551, 0.294)	0.12	-0.420 (-1.841, 1.002)	0.563	-0.611 (-2.039, 0.817)	0.401	-1.616 (-3.053, -0.179)	0.028*	0.205
	1.129 (-0.294, 2.551)	0.12	Ref	Ref	0.709 (-0.711, 2.130)	0.328	0.518 (-0.904, 1.939)	0.475	-0.487 (-1.914, 0.939)	0.503	
	0.420 (-1.002, 1.841)	0.563	-0.709 (-2.130, 0.711)	0.328	Ref	Ref	-0.192 (-1.614, 1.231)	0.792	-1.196 (-2.625, 0.232)	0.101	
	0.611 (-0.817, 2.039)	0.401	-0.518 (-1.939, 0.904)	0.475	0.192 (-1.231, 1.614)	0.792	Ref	Ref	-1.005 (-2.427, 0.417)	0.166	
	1.616 (0.179, 3.053)	0.028*	0.487 (-0.939, 1.914)	0.503	1.196 (-0.232, 2.625)	0.101	1.005 (-0.417, 2.427)	0.166	Ref	Ref	
ALT (IU/L)	Ref	Ref	-0.561 (-2.336, 1.213)	0.535	-0.542 (-2.315, 1.230)	0.549	0.946 (-0.835, 2.727)	0.298	0.925 (-0.867, 2.718)	0.312	0.247
	0.561 (-1.213, 2.336)	0.535	Ref	Ref	0.019 (-1.753, 1.791)	0.983	1.507 (-0.266, 3.280)	0.096	1.487 (-0.292, 3.266)	0.101	
	0.542 (-1.230, 2.315)	0.549	-0.019 (-1.791, 1.753)	0.983	Ref	Ref	1.488 (-0.286, 3.262)	0.1	1.468 (-0.314, 3.249)	0.106	
	-0.946 (-2.727, 0.835)	0.298	-1.507 (-3.280, 0.266)	0.096	-1.488 (-3.262, 0.286)	0.1	Ref	Ref	-0.020 (-1.794, 1.754)	0.982	
	-0.925 (-2.718, 0.867)	0.312	-1.487 (-3.266, 0.292)	0.101	-1.468 (-3.249, 0.314)	0.106	0.020 (-1.754, 1.794)	0.982	Ref	Ref	
WBC (× 10^3^/µL)	Ref	Ref	0.221 (0.082, 0.361)	0.002**	-0.027 (-0.167, 0.112)	0.698	0.288 (0.149, 0.428)	< 0.001**	0.362 (0.222, 0.503)	< 0.001**	< 0.001**
	-0.221 (-0.361, -0.082)	0.002**	Ref	Ref	-0.249 (-0.388, -0.110)	< 0.001**	0.067 (-0.072, 0.206)	0.345	0.141 (0.001, 0.281)	0.048*	
	0.027 (-0.112, 0.167)	0.698	0.249 (0.110, 0.388)	< 0.001**	Ref	Ref	0.316 (0.177, 0.455)	< 0.001**	0.390 (0.250, 0.530)	< 0.001**	
	-0.288 (-0.428, -0.149)	< 0.001**	-0.067 (-0.206, 0.072)	0.345	-0.316 (-0.455, -0.177)	< 0.001**	Ref	Ref	0.074 (-0.065, 0.213)	0.298	
	-0.362 (-0.503, -0.222)	< 0.001**	-0.141 (-0.281, -0.001)	0.048*	-0.390 (-0.530, -0.250)	< 0.001**	-0.074 (-0.213, 0.065)	0.298	Ref	Ref	

*Note* The p-value represents the significance of coefficients in (a) TE group, (b) AE only group, (c) RE only group, (d) insufficient group (e) inactive group compared to reference group; (f) The p-value for the global test to confirm if there is at least one difference among the groups; * *p* < 0.05; ** *p* < 0.01

*Abbreviation* AE, aerobic exercise; Alt, alanine aminotransferase; AST, aspartate aminotransferase; BMI, body mass index; BP, blood pressure; CI, confidence interval; HbA1c, glycated hemoglobin; HDL, high-density lipoprotein; HOMA-IR, homeostasis model assessment-estimated insulin resistance; LDL, low-density lipoprotein; RE, resistance exercise; TE, total exercise; TG, triglyceride; TyG index, triglyceride and glucose index; WBC, white blood cell; WC, waist circumference

**Fig. 2:**
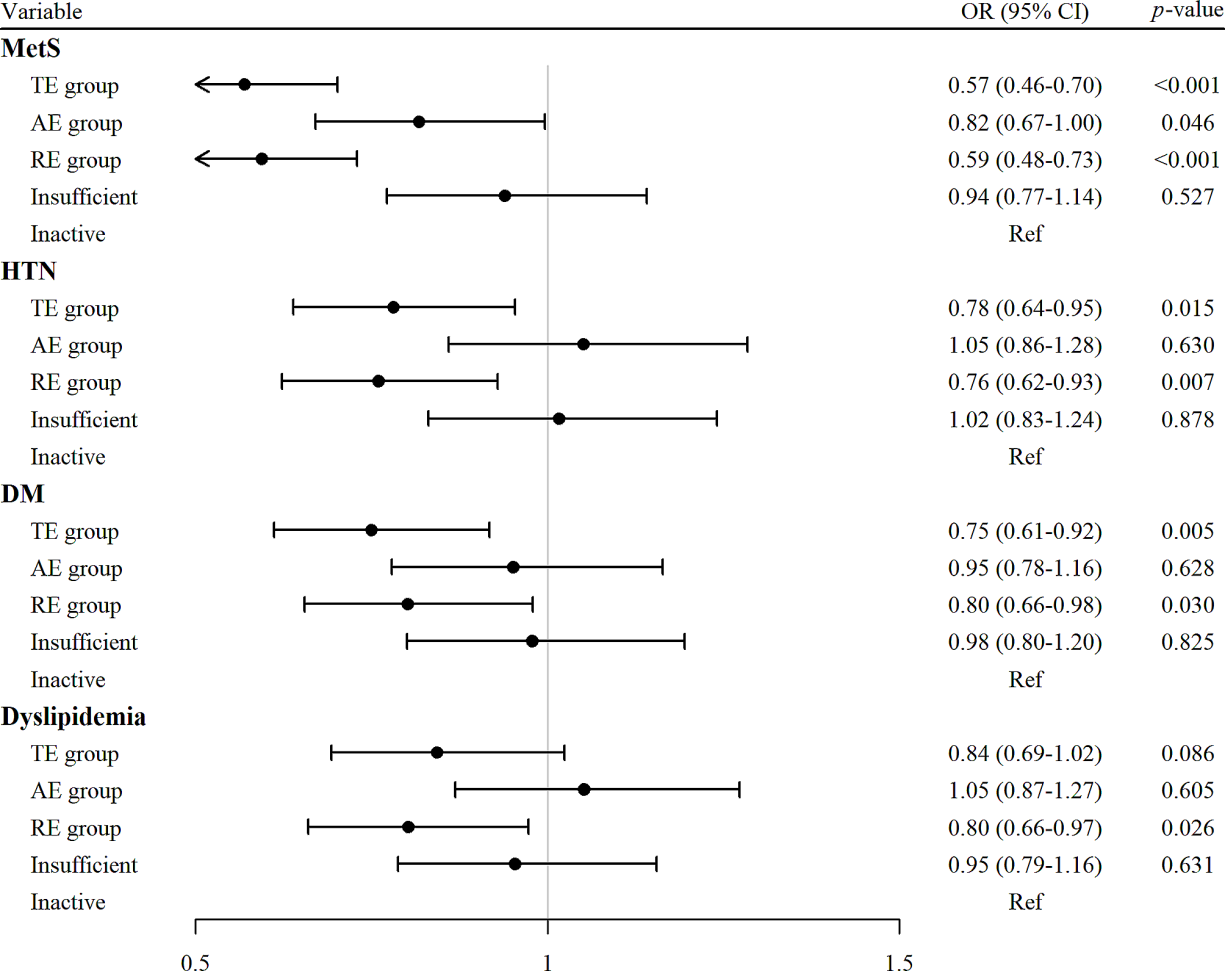
Forest plot for comparison of cardiometabolic disorders between five types of physical activity. MetS, Metabolic syndrome; HTN, Hypertension; DM, Diabetes mellitus; AE, aerobic exercise; RE, resistance exercise; TE, total exercise; OR, odds ratio; 95% CI, 95% confidence interval

Table 3 presents the characteristics of the AE and RE groups. Before PSM, the AE group exhibited a lower average age, a higher proportion of females, and lower rates of smoking than the RE group. After PSM using exact age and sex matching, in contrast to AE group, the RE group showed a lower prevalence of BMI-defined obesity and smaller WC values. Furthermore, mean BP, total cholesterol, fasting glucose, HbA1c, TyG index, and WBC count were also statistically lower in the RE group. The prevalence of cardiometabolic diseases (MetS, HTN, DM, and dyslipidemia) showed no significant difference before PSM, but after matching RE group showed lower percentages than AE group in all diseases.

**Table 3:** Clinical characteristics in the AE and RE groups pre- and post-PSM

Variable	Pre-PSM				Post-PSM			
	Overall	AE group	RE group	*p*-value	Overall	AE group	RE group	*p*-value
Unweighted N	3,847	1,983	1,864		2,920	1,460	1,460	
Age (years)	47.15 ± 16.06	45.36 ± 15.22	49.11 ± 16.72	< 0.001**	51.17 ± 16.22	51.17 ± 16.22	51.17 ± 16.22	> 0.999
Sex (%)				< 0.001**				> 0.999
Male	56.5	49.7	63.8		51.6	51.6	51.6	
Female	43.5	50.3	36.2		48.4	48.4	48.4	
Educational status (%)				0.047*				0.165
Elementary school	7.8	7	8.8		11.6	12.9	10.4	
Middle school	7.1	6.3	8		9.9	9.5	10.3	
High school	37.5	37.6	37.3		35.4	35.6	35.1	
College or University	47.6	49.1	46		43.1	42	44.2	
Alcohol consumption (yes, %)	60.2	60.1	60.2	0.932	55.4	56.3	54.6	0.372
Smoking history (%)				< 0.001**				0.782
No	53.7	57.1	50.1		56.4	56.2	56.7	
Current	18.2	19.3	17		16.4	16.9	16	
Former	28.1	23.7	33		27.1	26.9	27.3	
Household income status (%)				0.209				0.443
Low	12	10.8	13.2		15.1	15.8	14.5	
Mid-low	21.8	21.9	21.7		23.4	23.8	23	
Mid-high	29	30.1	27.8		27.7	28.1	27.3	
High	37.2	37.2	37.2		33.8	32.4	35.1	
Body mass index (%)				0.002**				< 0.001**
Underweight (< 18.5)	3.3	3.6	3		3.4	3.2	3.6	
Normal (≥ 18.5 and < 25)	58.4	55.4	61.8		60.7	56	65.3	
Obese (≥ 25)	38.3	41.1	35.2		35.9	40.8	31.1	
WC (cm)	84.20 ± 10.66	84.37 ± 10.96	84.01 ± 10.32	0.377	84.12 ± 10.47	85.17 ± 10.57	83.07 ± 10.27	< 0.001**
Mean BP (mmHg)	89.55 ± 10.47	89.73 ± 10.88	89.34 ± 10.01	0.299	90.03 ± 10.50	90.87 ± 10.90	89.19 ± 10.02	< 0.001**
Total cholesterol (mg/dL)	193.41 ± 38.03	195.59 ± 38.59	191.02 ± 37.26	0.001**	193.13 ± 38.94	194.62 ± 40.03	191.65 ± 37.78	0.039*
TG (mg/dL)	129.74 ± 116.10	131.58 ± 123.47	127.73 ± 107.46	0.356	129.82 ± 124.49	133.70 ± 134.29	125.95 ± 113.77	0.093
HDL-cholesterol (mg/dL)	52.61 ± 13.06	53.22 ± 13.21	51.95 ± 12.86	0.008**	52.91 ± 13.27	53.01 ± 13.36	52.82 ± 13.18	0.701
LDL-cholesterol (mg/dL)	116.37 ± 33.68	117.64 ± 34.00	114.98 ± 33.27	0.03*	115.81 ± 34.21	116.43 ± 35.12	115.19 ± 33.27	0.325
Glucose (mg/dL)	99.67 ± 20.31	99.80 ± 21.52	99.52 ± 18.90	0.693	100.80 ± 21.43	102.05 ± 22.89	99.54 ± 19.80	0.002**
Insulin (IU/L)	8.83 ± 6.42	9.06 ± 6.42	8.57 ± 6.40	0.039*	8.66 ± 6.57	8.86 ± 6.24	8.45 ± 6.89	0.093
HOMR-IR	2.24 ± 1.90	2.30 ± 1.91	2.18 ± 1.89	0.091	2.22 ± 1.98	2.29 ± 1.89	2.16 ± 2.07	0.064
HbA1c (%)	5.72 ± 0.74	5.72 ± 0.79	5.71 ± 0.69	0.739	5.78 ± 0.75	5.83 ± 0.79	5.74 ± 0.70	0.001**
TyG Index	8.56 ± 0.66	8.57 ± 0.66	8.55 ± 0.65	0.459	8.57 ± 0.65	8.61 ± 0.65	8.53 ± 0.64	< 0.001**
AST (IU/L)	25.33 ± 17.50	24.82 ± 13.60	25.88 ± 20.94	0.145	25.30 ± 15.45	25.24 ± 12.10	25.35 ± 18.20	0.857
ALT (IU/L)	24.85 ± 20.54	24.83 ± 19.38	24.88 ± 21.74	0.958	23.94 ± 18.53	24.36 ± 17.60	23.52 ± 19.42	0.223
WBC (× 10^3^/µL)	6.10 ± 1.63	6.17 ± 1.64	6.03 ± 1.60	0.031*	6.03 ± 1.61	6.15 ± 1.64	5.91 ± 1.57	< 0.001**
MetS (yes, %)	25.2	26.1	24.1	0.198	26.6	30	23.2	< 0.001**
HTN (yes, %)	53.1	51.8	54.6	0.14	57.3	60.1	54.5	0.002**
DM (yes, %)	51.6	50.9	52.2	0.522	56.8	58.8	54.7	0.028*
Dyslipidemia (yes, %)	33.2	33.5	32.9	0.748	36.1	38.5	33.6	0.007**

*Note* All continuous data are presented as means ± standard deviation (SD); All categorical data are presented as percentage (%); †Student’s t-test or Pearson’s chi-square test were used to compare the AE and RE groups; * *p* < 0.05; ** *p* < 0.01

*Abbreviation* AE, aerobic exercise; ALT, alanine aminotransferase; AST, aspartate aminotransferase; BMI, body mass index; BP, blood pressure; DM, diabetes mellitus; HDL, high-density lipoprotein; HOMA-IR, homeostasis model assessment-estimated insulin resistance; HTN, hypertension; LDL, low-density lipoprotein; MetS, metabolic syndrome; PSM, propensity score matching; RE, resistance exercise; TG, triglyceride; TyG index, triglyceride and glucose index; TE, total exercise; WC, waist circumference

**Fig. 3:**
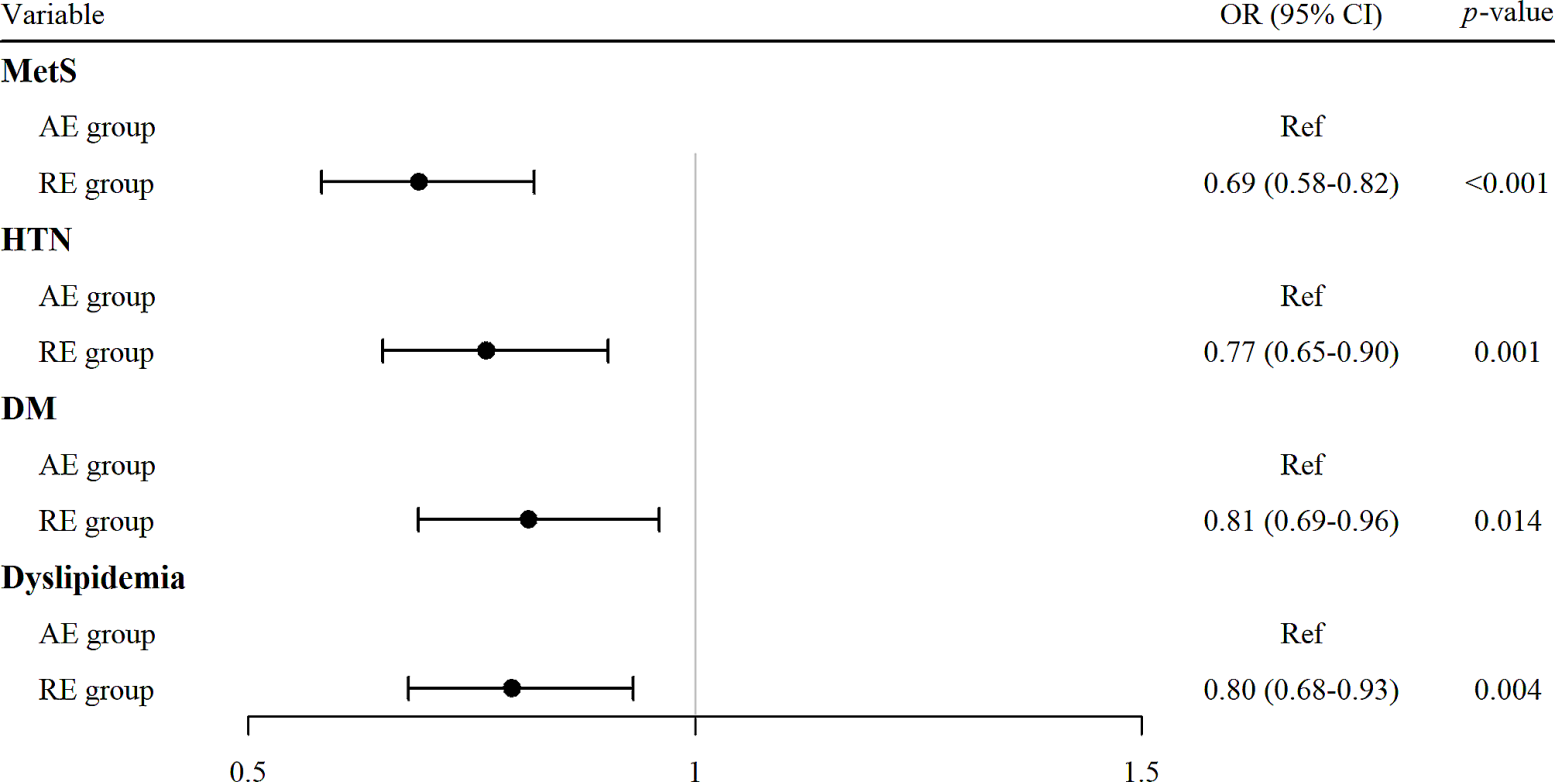
Forest plot for comparison of cardiometabolic disorders between AE and RE. MetS, Metabolic syndrome; HTN, Hypertension; DM, Diabetes mellitus; AE, aerobic exercise; RE, resistance exercise; TE, total exercise; OR, odds ratio; 95% CI, 95% confidence interval

Table 4 shows the regression coefficients of the biochemical and metabolic indices of the RE group with the AE group set as a reference after PSM. Compared with the AE group, the RE group showed significantly lower BMI and WC, mean BP, total cholesterol, glucose, HbA1c, TyG index, and WBC count. Insulin level and HOMA-IR showed a lower trend in RE as well, although not statistically significant. HDL-C, LDL-C, and TG levels showed no significant differences between the two groups.

**Table 4:** Comparison between AE and RE post-PSM

Variable	AE group	RE group	
		Coefficient (95% CI)	*p*-value
BMI (kg/m^2^)	Ref	-0.658 (-0.907 - -0.408)	< 0.001**
WC (cm)	Ref	-2.039 (-2.705 - -1.373)	< 0.001**
Mean BP (mmHg)	Ref	-1.677 (-2.404 - -0.950)	< 0.001**
Total cholesterol (mg/dL)	Ref	-3.098 (-5.895 - -0.301)	0.03*
TG (mg/dL)	Ref	-7.148 (-15.984–1.688)	0.113
HDL-cholesterol (mg/dL)	Ref	-0.182 (-1.061–0.696)	0.684
LDL-cholesterol (mg/dL)	Ref	-1.443 (-3.909–1.023)	0.251
Glucose (mg/dL)	Ref	-2.433 (-3.922 - -0.945)	0.001**
Insulin (IU/L)	Ref	-0.407 (-0.882–0.067)	0.092
HOMA-IR	Ref	-0.135 (-0.278–0.008)	0.064
HbA1c (%)	Ref	-0.086 (-0.136 - -0.035)	< 0.001**
TyG index	Ref	-0.079 (-0.123 - -0.035)	< 0.001**
AST (IU/L)	Ref	0.111 (-0.999–1.221)	0.845
ALT (IU/L)	Ref	-0.860 (-2.162–0.443)	0.196
WBC (× 10^3^/µL)	Ref	-0.218 (-0.328 - -0.108)	< 0.001**

*Note* †The p-value represents the significance of coefficient in RE group compared to AE group; * *p* < 0.05; ** *p* < 0.01

*Abbreviation* AE, aerobic exercise; ALT, alanine aminotransferase; AST, aspartate aminotransferase; BMI, body mass index; BP, blood pressure; CI, confidence interval; HDL, high-density lipoprotein; HOMA-IR, homeostasis model assessment-estimated insulin resistance; HTN, hypertension; LDL, low-density lipoprotein; PSM, propensity score matching; RE, resistance exercise; TG, triglyceride; TyG index, triglyceride and glucose index; WBC, white blood cell; WC, waist circumference

In Fig. 2 the ORs for cardiometabolic disorders are shown, with the inactive group set as a reference following PSM. MetS risk was lowest in TE group (OR 0.57, 95% CI 0.46–0.70, p-value < 0.001), closely followed by RE group (OR 0.59, 95% CI 0.48–0.73, p-value < 0.001). AE group also showed a slightly lower risk than the inactive group (OR 0.82, 95% CI 0.670–0.996, p-value = 0.046). HTN (TE: OR 0.78, 95% CI 0.64–0.95, p-value = 0.015, RE: OR 0.76, 95% CI 0.62–0.93, p-value = 0.007) and DM risk (TE: OR 0.75, 95% CI 0.61–0.92, p-value = 0.005, RE: OR 0.80, 95% CI 0.66–0.98, p-value = 0.03) were likewise lowest in the TE and RE groups (with no statistically significant differences between the two groups), while AE and insufficient group showed no significant risk reduction compared to the inactive group. Dyslipidemia risk was lowest in the RE group (OR 0.80, 95% CI 0.66–0.97, p-value = 0.026).

Fig. 3 shows the ORs of cardiometabolic disorders in the RE group compared with that in the AE group. All diseases showed lower risk in the RE group, as follows: MetS (OR 0.69, 95% CI 0.58–0.82, p-value < 0.001), HTN (OR 0.77, 95% CI 0.65–0.90, p-value = 0.001), DM (OR 0.81, 95% CI 0.69–0.96, p-value = 0.014), and dyslipidemia (OR 0.80, 95% CI 0.68–0.93, p-value = 0.004).

## Discussion

We aimed to determine the most effective exercise modality for reducing cardiometabolic risk in a Korean adult population. In the TE group, various metabolic risk markers, including WC, BP levels, glucose and insulin-related indices, and WBC counts, were shown to be significantly lower than those in the AE, insufficient, and inactive groups. The RE group showed a similar trend in values with TE compared to the other three groups. Notably, the TE group exhibited significantly higher HDL-C levels and lower TG/insulin-related levels compared with all other PA groups. Participants in the TE group had the lowest ORs for MetS. The risk of HTN and DM decreased in the TE group exclusively followed by RE group. In a comprehensive analysis comparing AE and RE, RE consistently showed advantages of lower BMI, WC, mean BP, total cholesterol and lower glucose, HbA1c, and WBC counts. Furthermore, RE was associated with lower risk of MetS, HTN, DM, and dyslipidemia when compared with AE. However, prospective studies with larger databases are needed to definitively infer the cause and effect of our findings.

It has been widely acknowledged that exercise and lifestyle play pivotal roles in preventing and managing cardiometabolic diseases, and related mortality [14–16]. Numerous studies have shown that combining aerobic and resistance exercises is more effective than aerobic or resistance exercises alone in improving parameters such as fat mass, metabolic profiles, and inflammatory markers [17–21]. Concurrent exercise holds the potential to affect diverse metabolic pathways synergistically. For instance, AE promotes increased aerobic capacity, which involves central adaptations and metabolic changes in skeletal muscle, such as heightened mitochondrial density and capillarization [22]. In addition, it is well known that aerobic exercise can improve insulin resistance by increasing insulin signaling proteins such as GLUT4 and GLUT4 vesicle-associated protein. Because insulin resistance is the key factor of cardiometabolic syndrome, it is obvious the effect of aerobic exercise in improving insulin resistance is expected to treat cardiometabolic syndrome [23]. Conversely, RE leads to muscle hypertrophy, increased strength and power [24], and potential improvements to bone mineral density [25]. RE can promotes muscle growth by stimulating IGF-1, which is well known for synthesizing protein in skeletal muscle and promoting body growth [26]. About 40% of total body mass of human consists of skeletal muscle and the skeletal muscle is the main engine of insulin-mediated glucose uptake and oxidation of fatty acid [27]. As RE improve muscle growth, glucose uptake and fatty acid oxidation by insulin signaling may be accelerated. Consistent with prior studies, our findings strongly suggest that integrating aerobic and resistance training into a comprehensive exercise program synergistically reduces the risk of chronic non-communicable diseases.

Nonetheless, previous studies have predominantly focused on evaluating the effectiveness of AE, leaving a notable gap in evidence concerning the effectiveness of RE, whether conducted independently or in combination with AE, in relation to cardiometabolic risk. Similar to AE, RE offers benefits for multiple cardiometabolic risk factors, including improved insulin sensitivity and glycemic control [28], positively affecting factors such as glycogen synthase activity, glucose transporter type 4 protein content, and AMP-activated protein kinase [5, 29]. Additionally, RE contributes to cardiometabolic prevention through enhancing weight management, endothelial function, and hemodynamics [30–33]. In accordance with previously reported findings, our study findings suggest that RE as well as AE in mitigates metabolic abnormalities such as obesity, HTN, dyslipidemia, CMS, and NAFLD during adulthood; however, RE surpassing AE differ from some previous investigations [34–36]. Most recently, a large-scale meta-analysis of 270 randomized controlled trials observed RE to be slightly more antihypertensive than AE which supports our findings [37]. The precise cause and mechanism underlying the disparities in findings between our study and some others remain uncertain. Variations in sample sizes and differences in the types and intensities of exercises could account for the variations in the estimated effects of RE and AE. Furthermore, ethnic differences may explain the divergent outcomes observed in various studies. In contrast with Western populations, Asian populations present with lower levels of muscle mass, diminished beta cell functional capacity, and heightened insulin resistance [38]. These factors likely contribute to ethnic disparities in terms of exercise efficacy and susceptibility to metabolic complications [39, 40]. Additional extensive cohort studies and well-designed clinical trials are imperative to elucidate these complexities and establish the optimal exercise regimen for cardiometabolic prevention.

This study has some strengths. In relation to current understanding, it innovatively suggests that RE could be related with enhanced advantages over AE in addressing chronic metabolic diseases among the adult population. Additionally, we conducted an analysis using a 1:1 PSM approach to reduce baseline group differences, used mainly when randomized controlled trials are not feasible, and employed a well-known database that accurately represents the Korean population.

Our study had several limitations. First, the cross-sectional study design cannot be used to determine causality. Second, our results were limited to a single ethnic group; therefore, it is difficult to generalize our findings to other populations. Third, our study did not consider essential dietary factors among the various lifestyle aspects that can influence the effect of exercise and cardiometabolic risk. Last, we relied on self-reported questionnaires for evaluating PA, which may have influenced our results in terms of comparing AE and RE and their effects on metabolic parameters.

In conclusion, AE and RE in combination was found to show the strongest association with cardiometabolic risks in Korean adults. Importantly, our study indicates that RE might confer a more favorable cardiometabolic effect than AE. Considering the more effective exercise modality for different medical conditions, additional research is warranted to validate these advantageous exercise effects.

## Data Availability

The datasets analysed during the current study are publicly available in the KNHANES repository, at (https://knhanes.cdc.go.kr/main.do).
